# Supplementary material for: The interactome of intact mitochondria by cross-linking mass spectrometry provides evidence for coexisting respiratory supercomplexes
Source: Mol Cell Proteomics. 2017 Dec 8;17(2):216–32. doi: 10.1074/mcp.RA117.000470 (PMC5795388; doi:10.1074/mcp.RA117.000470)
Supplement: Supplemental Data [file supp_RA117.000470_133922_0_supp_23978_fzffwf.zip › spectra_annotation/mito_DR_spectra_annotation/466-1-40-1-6-1.pdf]

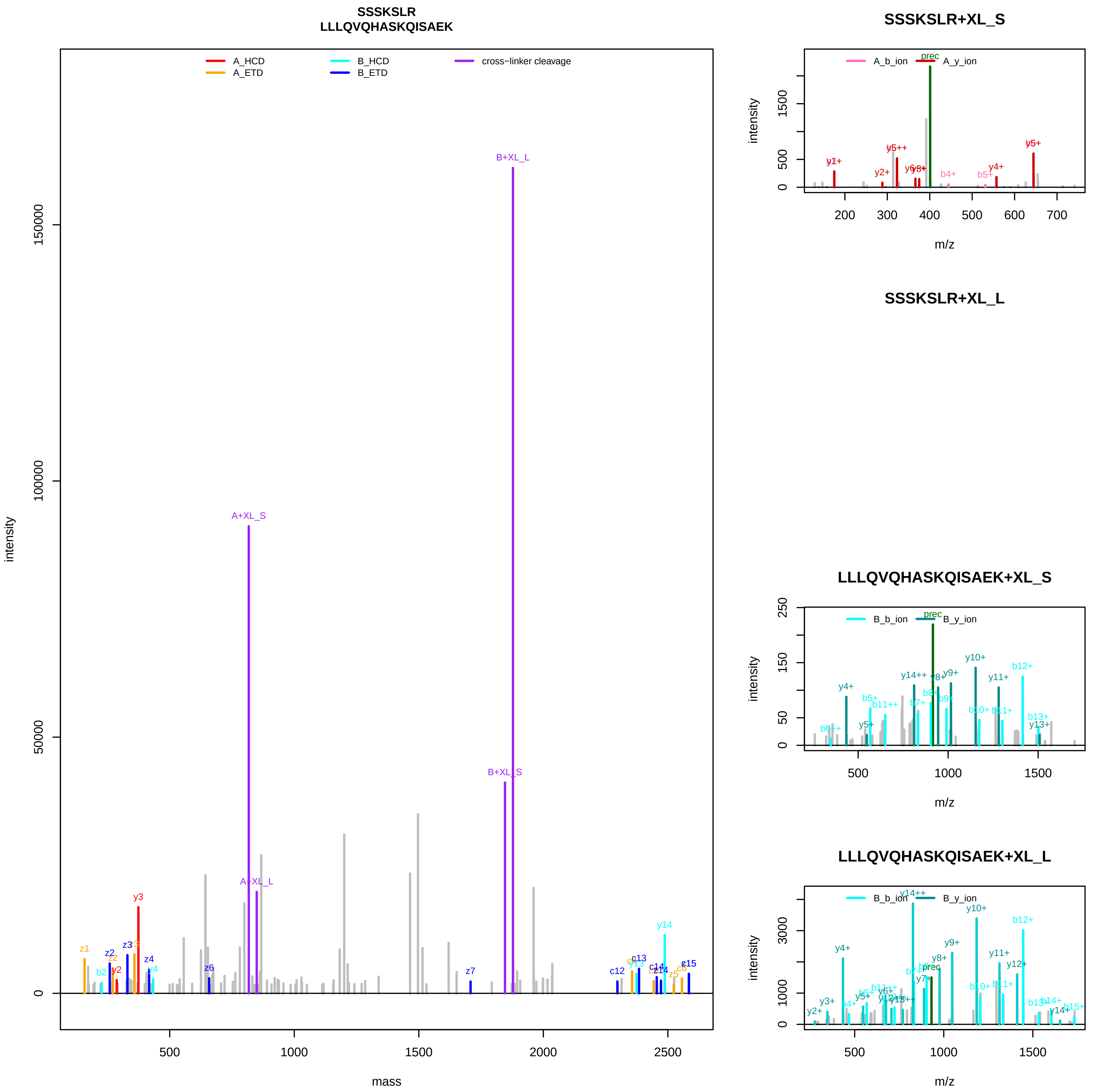
SSSKSLR
LLLQVQHASKQISAEK
A_HCD
A_ETD
B_HCD
B_ETD
cross−linker cleavage
intensity
mass
0
50000
100000
150000
500
1000
1500
2000
2500
B+XL_L
A+XL_S
B+XL_S
A+XL_L
y3
z1
b2
z2
y2
z3
z4
y4
z6
z7
c12
c13
y13
c14
z14
y14
z5
c6
z15
SSSKSLR+XL_S
A_b_ion
A_y_ion
prec
y1+
y2+
y5++
y6+
y8+
b4+
b5+
y4+
y5+
m/z
SSSKSLR+XL_L
LLLQVQHASKQISAEK+XL_S
B_b_ion
B_y_ion
y4+
y5+
b5+
b11++
y14++
y8+
y9+
y10+
y11+
b12+
b7+
b8+
b9+
b10+
b11+
b13+
y13+
LLLQVQHASKQISAEK+XL_L
y2+
y3+
y4+
y5+
y6+
y7+
y8+
y9+
y10+
y11+
y12+
y13++
y14++
y14+
b10+
b11+
b12+
b13+
b14+
b15+
